# Litigation Cases After Post-Bariatric Surgery: Lesson from the Past

**DOI:** 10.1007/s00266-023-03287-1

**Published:** 2023-02-23

**Authors:** Federico Facchin, Andrea Pagani, Filippo Andrea Giovanni Perozzo, Carlotta Scarpa, Franco Bassetto, Vincenzo Vindigni

**Affiliations:** 1 Plastic and Reconstructive Surgery Unit, Department of Neurosciences, University of Padua, Via Giustiniani 2, 35128 Padua, Italy; 2 Plastic Surgery Unit, San Bortolo Hospital, 36100 Vicenza, Italy; 3 Clinic and Polyclinic of Plastic and Hand Surgery, Technical University of Munich, 81675 Munich, Germany

**Keywords:** Massive weight loss, Post-bariatric surgery, Body contouring procedures, Post-operative complications, Legal litigation, Medico-legal issues

## Abstract

**Background:**

Due to the high demand of post-bariatric surgeries, the number of litigation cases is rapidly growing. Even if surgical mistakes still represent one of the main causes of medico-legal issues, many disputes depend on what happens in the post-operative course. In this article we analyzed the litigation cases that occurred in our Plastic Surgery Department, the current literature about medico-legal disputes and the importance of the doctor–patient relationship.

**Patients and methods:**

The medical records of 788 post-bariatric surgeries, the post-operative complications and the related litigation cases from January 2015 to December 2019 were collected, analyzed and compared.

**Results:**

We performed 380 abdominoplasties, 28 torsoplasties, 65 breast reductions, 99 mastopexies, 94 brachioplasties, 52 thighplasties, 65 liposuctions and 5 facelifts between 2015 and 2019. Eight patients complained of medical issues and claimed for litigation. Despite in all cases the judges highlighted the risk of consent misinterpretation, the payout was granted only in one case.

**Conclusion:**

Post-bariatric patients often mistake their preoperative condition and consider body contouring procedures as an aesthetic surgery treatment. Patients should be therefore clearly informed about the complexity of body contouring procedures after massive weight loss, which should never be compared to aesthetic surgery. Surgeons should always promote the communication with their patients and build a strong and trustworthy relationship. This attitude will allow to deal more easily with complications and, in the worst situations, with medico-legal litigations.

**Level of Evidence IV:**

This journal requires that authors assign a level of evidence to each article. For a full description of these Evidence-Based Medicine ratings, please refer to the Table of Contents or the online Instructions to Authors www.springer.com/00266.

## Introduction

Obesity is a multifactorial health disease affecting the adult American population with 41.9% of obese and 9.2% of morbidly obese patients [1]. Overweight patients are at major risk for developing type 2 diabetes, cardiovascular and gastrointestinal diseases, joint and muscular disorders, respiratory problems and certain types of cancer [2]. These conditions represent the leading causes of preventable death. In addition, the daily difficulties of obese patients alter significantly their quality of life, generating heavy psychological disorders. Nonetheless, drastic weight loss impacts severely patients’ health and well-being [3]. Massive Weight Loss (MWL) results, most of the time, in adipose-cutaneous excess and skin dystrophy causing pain and impaired quality of life [4–6].

Due to the increased frequency of obesity and the number of massive weight loss patients, the demand for body contouring procedures is rapidly growing [7, 8]. Plastic surgery plays a determinant role in improving patients’ quality of life, helping them to maintain their weight and shape [9]. According to the American Society of Plastic Surgeons (ASPS), 85% of MWL patients seek body contouring surgeries with a calculated number of 46.577 procedures in 2020 [10]. Because of their intrinsic clinical complexity, MWL patients can present insidious post-operative complications such as seroma, wound infection, dehiscence, necrosis, lymphorrhea, asymmetry and thrombosis. All these issues are very common with rates ranging from 8 to 66% [11–13]. Even nutritional deficiencies, anemia due to malabsorption and weight regain can significantly affect the post-operative results of body contouring surgeries [14, 15]. Whenever there is mention of post-operative complications, the dissatisfaction of the patient is around the corner with the consequent origin of medico-legal disputes. Like many other surgical fields, even post-bariatric surgery is exposed to medico-legal matter. Litigations are an important topic for our healthcare system with a significant impact on clinicians’ daily practice. Malpractice claims significantly affect medical costs and health care quality [16]. Litigations are slowly reshaping various medico-legal aspects of the doctor–patient relationship and, in many cases, the patient’s attitude toward the doctor.

In this article we establish the number, the causes and the impact of medico–legal disputes after 788 post-bariatric surgeries performed in our Plastic Surgery Department between 2015 and 2019. This to improve our daily clinical practice, patients’ consultation process and to find new ways to improve our medical performance. Finally, we aim to prevent the increase in medico-legal disputes in the future, communicating the achievable surgical results to patients with their original clinical condition always in mind.

## Materials and Methods

The study was conducted according to the human studies guidelines of our University Hospital in Padua (Italy) and with the World Medical Association Declaration of Helsinki (June 1964) and subsequent amendments.

After recording 788 post-bariatric procedures of our Department between January 2015 and December 2019, the number of major and minor complications and the total number of complications allowed us to establish the rate of post-operative complications at the Department of Plastic Surgery of our University Hospital. Exclusion criteria were incomplete records and failure to follow-up. In addition, a retrospective review of medico-legal issues presented to the same Department in the same period was performed with particular insight to patient demographics, procedures performed, causes and following injury of malpractice claim, verdict and eventual damage compensation in body contouring procedures. Finally, authors critically looked for a possible correlation between their surgical performance, the reported complications and the malpractice claims. This to find a causal nexus between surgery and litigation. Most of the time, the claim was the result of a misunderstanding between the surgeon and the patient during the preoperative consultation.

## Statistical Analysis

The data gathered were analyzed using IBM SPSS Statistics software. The quantitative variables that followed a normal distribution were summarized as means  ± standard deviations [SD), and medians and ranges were recorded for non-Gaussian variables. Qualitative variables were summarized by number and as percentage of cases. The paired t test or Wilcoxon rank test was used to study the differences between means while ANOVA or the Kruskal–Wallis test for comparing changes in variables with more than two categories. Statistical significance was set at *P* < 0.05.

## Results

A total number of 788 surgeries were performed between January 2015 and December 2019: 388 abdominoplasties, 28 torsoplasties, 65 reduction mammoplasties, 99 mastopexies with (79) or without (20) prosthesis, 94 brachioplasties, 52 thighplasties, 65 liposuctions and 5 facelifts. The medical records of the procedures were analyzed and evaluated. Before the body contouring operation, patients underwent different bariatric surgeries: 16.32% of patients underwent a gastric bypass, 7.14% a bandage, 67.34% a sleeve gastrectomy, 2% a balloon surgery and 7.2% underwent other procedures. According to the Pittsburgh rating scale, the majority of patients were graded 2 or 3 [17]. In total, 46% of subjects underwent a combined post-bariatric procedure, with the most frequent combination consisting in mastopexy and brachioplasty. Most patients who underwent a post-bariatric procedure were female (96%). Patient age ranged from 20 to 71 years (mean age 45.3 years). The average BMI before the body contouring procedure was 26.4 ± 4.1 kg/m^2^ (ranging from 20 to 42.9 kg/m^2^) with mean weight loss of 19.2 kg/m^2^ reached after surgeries. In total, 20.4% of patients had a smoking history. The average time from consultation to surgery was 136 days. The follow-up period ranges from 2.5 to 7.5 years. Corrective procedures were performed in 100 patients (12.69%).

A total number of 362 (46%) complications were found, 152 (19%) of which were described as major and 210 (27%) as minor complications (i.e., delayed healing, unfavorable scarring, hematoma and seroma). Indeed, thighplasty had the highest complications rate in our series (i.e., 63%). Data regarding surgeries are reported in Table 1.

**Table 1:** Type of procedures, major and minor complications with associated complication rates. Of 788 surgeries, we observed a total number of 362 complications (complication rate 46%). While 152 were major complications, 210 were minor complications

	Number of procedures	Major complications	Minor complications	Total complications	Complication rate (%)
Abdominoplasty	380	98	88	186	49
Torsoplasty	28	8	6	14	50
Reduction mammoplasty	65	12	14	26	40
Mastopexy	20	5	3	8	40
Mastopexy with prosthesis	79	15	16	31	39
Brachioplasty	94	10	37	47	50
Thighplasty	52	3	30	33	63
Liposuction	65	1	15	16	25
Facelift	5	0	1	1	20
Total	788	152	210	362	46

**Fig. 1:**
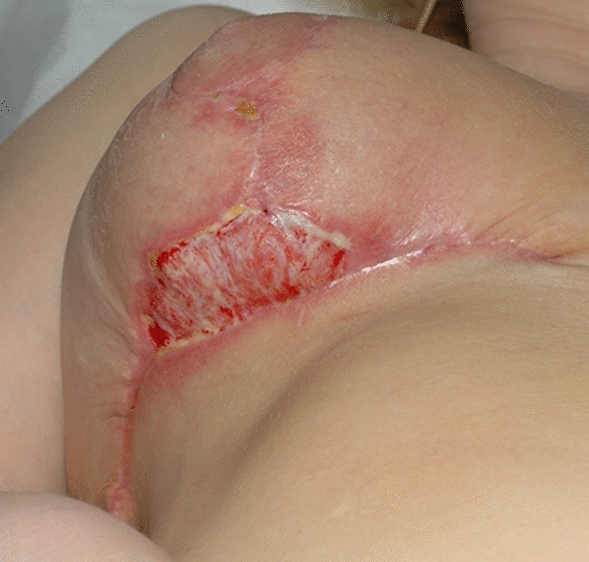
Wound dehiscence after reduction mammoplasty

**Fig. 2:**
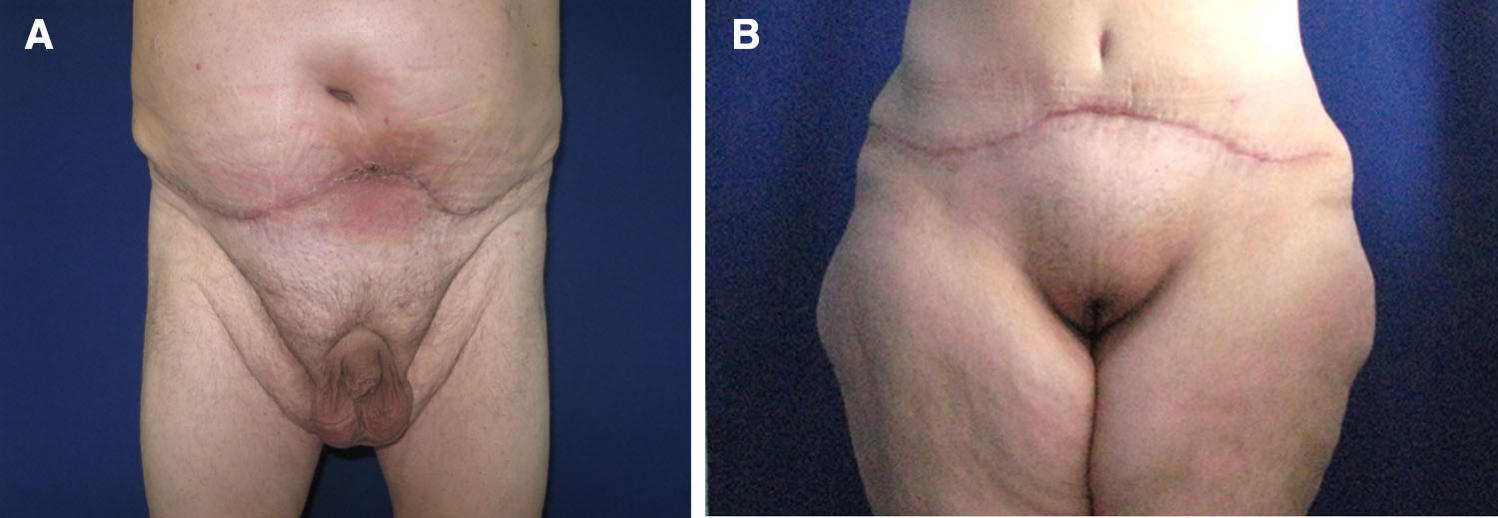
**a**, **b** Scar complications following abdominoplasty

Among almost 20,000 procedures performed in our Plastic Surgery Department between 2015 and 2019, from cosmetic to plastic and reconstructive surgery, the medical malpractice litigation cases were 42 (0.21%). Of all these surgeries, the total number of post-bariatric procedures were 788 (3.94%) and the related disputes were 8 (1.01%). Eight patients (1.01% of the total number treated with a body contouring procedure) pursued litigation claiming medical malpractice. Four patients after an abdominoplasty with a mean amount of removed tissue of 3.7 kg for every surgery. One patient after a reduction mammoplasty, two patients after a brachioplasty and one after a liposuction (Table 2). Three litigation cases were related to a major complication: relapse of diastasis recti with seroma in abdominoplasty, brachial plexus stupor in brachioplasty and skin necrosis in reduction mammoplasty (Fig. 1). The other five cases of litigation were based on minor complications, in particular on the alteration of cutaneous profile and bad scarring described as hypertrophic or too high scar placement (Fig. 2a, b).

**Table 2:** Litigation cases and rates after body contouring procedures. Of 788 surgical procedures performed between 2015 and 2019, there were 8 litigation cases, representing a litigation rate of 1,01% for body contouring procedures.

	Number of procedures	Litigation cases	Litigation rates (%)
Abdominoplasty	380	4	1.05
Torsoplasty	28	0	
Reduction mammoplasty	65	1	1.53
Mastopexy	20	0	
Mastopexy with prosthesis	79	0	
Brachioplasty	94	2	2.12
Thighplasty	52	0	
Liposuction	65	1	1.53
Facelift	5	0	
Total	788	8	1.01

Brachioplasty appeared to be the procedure more prone to litigation (2.12%) even without a statistical difference with the other procedures (*p *> 0.05). Patient characteristics showed no significant relation with preoperative BMI and weight loss. In only one of the eight litigation cases the patient was agreed, and a payout was granted. In the other disputes, the judge declared the correct behavior of the surgeon, accepting the differences of surgical outcomes and complications between aesthetic and post-bariatric procedures. Nevertheless, the examination of the informed consent highlighted the risk of possible misinterpretation of the final results related to the body contouring surgery.

## Discussion

Post-bariatric surgery helps to maintain a stable reduction of the BMI, stimulate patients’ sociability and is positively related to physical and psychological changes of MWL patients. To trigger these features, surgeons should consider every single medical aspect during hospitalization [18]. However, surgical complications following post-bariatric procedures are reported in nearly 46% of non-smokers and 69% of smokers [19]. Bad or asymmetrical scarring, wound dehiscence, necrosis, hematoma, seroma, lymphedema, neuropathy and deep venous thromboembolism can be developed during the hospital stay.

A proper preoperative counseling is key for a solid and faithful surgeon–patient relationship and can set a positive attitude between them. Despite patients and lawyers contest mainly the liability of medical actions, an incomplete informed consent is frequently the primary cause of medical issues [20]. The Report of the Court of Patient’s Rights of 2008 declared that only 67% of litigation cases were due to surgical mistakes, recognizing the importance of how information should be given to patients and the significance of the doctor–patient relationship [21]. The importance of setting clear standards during the preoperative consultation, productive communication, the explanation of risks, the functional goals, and the achievable cosmetic results in our surgeries should be the primary main goal for a surgeon. Even though the ideal instrument to obtain a precise preoperative surgical risk assessment is still lacking, a thorough discussion with patients should be aimed in identifying their expectation and their understanding about surgical risks. Informed consent is essential in case of an undesired outcome: patients can better deal with the complication, accepting a non-perfect outcome, if they are well informed. Moreover, an irreproachable surgical technique has to be done despite the higher risk of complications for these procedures. A total understanding of the differences between expectations and reality is needed. This to enhance patients’ education and improve shared medical decision making. Even for this reason, the Italian Society of Plastic, Reconstructive and Aesthetic Surgery (SICPRE] has recently standardized a detailed consent for surgeons and patients [22]. Nonetheless, patient selection has a crucial role in the healing process, considering that a higher complications risk has been connected to patients’ past medical history and habitus (i.e., the difference of their BMI before and after contouring, diabetes, hypertension, nutritional deficits and smocking status). Uncontrolled diabetes or active smocking status can be considered contraindication for the surgery [23–25].

From the legal point of view, MWL patients should not be compared to aesthetic elective patients who seek cosmetic improvements. Whereas MWL patients require surgery to improve their clinical condition after significant para-physiologic changes due to their previous obese status (from poor skin quality to alteration of vascular structure [26, 27]), the aesthetic patient is healthy and wants to improve its body shape. However, in front of the law, the need to undergo a plastic surgery treatment oftentimes equates the MWL patient to the aesthetic patient. Despite the name of body contouring surgeries is the same as that of aesthetic procedures, the initial clinical condition of MWL patients compared to aesthetic patients is extremely different with a higher risk for complications, due to a poor skin quality of MWL patients, their nutritional deficits, comorbidities and a greater extent of incisions. The need for extensive body exposure and intraoperative patient repositioning increases the risk of hypothermia and its associated complications [28]. In addition, the duration of surgery plays also an important role. Concerning this point, the advantages of performing combined procedures during the same surgical session are still debatable due to the potential increment of complications [29–32].

It is therefore necessary to differentiate these two categories from a legal point of view, in order to judge correctly those patients who appeal to the lawyer and those doctors who are called to defend themselves.

The most common reasons for litigation in the literature are the absence of a valid informed consent and post-operative cosmetic deformities due to mismanagement of patient’s expectations [33]. In general, the low level of disputes in our Department indicates the high standard of healthcare offered, even with the higher number of disputes after body contouring treatments (1.01%). Nevertheless, the minimal payment accorded with the patients could relate to a high expectation of post-bariatric patients. In contrast to our data, the analysis of 253 studies performed by Hasanbegovic et al. showed that although the complication rates in post-bariatric patients are 60–87% more common than in non-bariatric patients, the litigation cases arising in post-bariatric patients is lower than in the non-bariatric patients [4].

Regarding the type of surgery, breast augmentation and breast reduction represent the most common fields of litigation with 39.4% and 37.7% of cases, respectively. According to a recent review, breast reduction is considered the procedure with the most litigation in the NHS due to poor cosmetic results with £ 38.000 average payout. Relevant causes of unsatisfaction according to the patients are poor cosmetic results and scarring [33].

Extensive scarring is also an undesirable topic of litigation and a successful outcome is strongly desired by patients. While trunk scars should be transverse, symmetrical, flat and well-hidden under standard undergarments, the brachioplasty and vertical medial thigh scars should lie medially and closely drift from posterior to mid medial extremity. Several dressing devices have shown improvement in wound healing and scarring [34]. Nevertheless, scarring is most of the time considered as a minor complication rather than a surgical mistake and is not considered an unsolvable problem. Still, the literature confirms the importance of informed consent and of truthful discussion about the specific treatment, alternatives, complications and surgery results [35–37]. In our study, the average time from the consultation until the surgery was 136 days.

The Pittsburgh Rating Scale is the reference classification in post-bariatric patients and classifies patient deformities following weight loss in a clinically useful way. However, this classification does not compare patients before and after surgery. It would be useful to have an objective numerical classification able to compare the preoperative status of each individual patient with the final outcome of each single operation in order to anticipate a priori the surgical results and quantify the surgical improvement. A new classification system could also be of interest for judges to evaluate patients using a numerical index.

Performing the study in an academic center could underestimate the number of medico-legal issues especially if compared to private practice. On the contrary, unfortunately in our country, many law firms support patients to submit a claim against public institutions looking for an easier source of payout [21].

## Conclusion

Altogether, even without an objective aesthetic damage, post-bariatric patients could misjudge their preoperative condition and risks, basing the litigation on the aesthetic outcome. Patients should be clearly informed about the complexity of body contouring procedures after massive weight loss which should never be considered and defined as an aesthetic procedure. As surgeons we have to enhance the relationship with our patients, the communication with them and give the right input to other surgeons to find new ways to improve their medical performances. A numerical pre- and post-operative assessment scale should be developed in order to objectively analyze the results and the improvements reached after surgery. Once these features have been achieved, surgeons will be able to deal more easily with post-operative complications and, in the worst scenario, with the consequent medico-legal litigations.
